# A New Perspective for Isolated Coronary Artery Ectasia: Cystatin C

**DOI:** 10.7759/cureus.11053

**Published:** 2020-10-20

**Authors:** Alper Karakus, Ahmet Tütüncü, Sencer Çamcı, Berat Uğuz, Gökhan Özmen, Hasan Arı, Mehmet Demir

**Affiliations:** 1 Department of Cardiology, Bursa Postgraduate Hospital, Bursa, TUR; 2 Department of Cardiology, Bursa Postgraduate Hospıtal, Bursa, TUR; 3 Department of Cardiology, Bursa Postgarduate Hospital, Bursa, TUR

**Keywords:** dilating vascular disease, coronary ectasia, endothelial dysfunction, positive arterial remodeling, aneurysm

## Abstract

Introduction

The pathophysiology of isolated coronary artery ectasia (iCAE) has not been clearly identified, although multiple abnormalities, including arteritis, endothelial dysfunction, and vascular destruction, have been reported. In this study, we aimed to analyze serum cystatin C concentrations in patients with iCAE and controls.

Methods

Forty-seven patients with iCAE (mean age: 55.9 ± 11.5) and 32 individuals with normal coronary angiography (mean age: 57.8.1 ± 9.6) were included in the study. Plasma cystatin C levels were measured by using the principle of particle-enhanced turbidimetric immunoassay (PETIA).

Results

Serum cystatin C concentrations were significantly lower in patients with iCAE compared with the control group (0.98 ± 0.17 mg/L versus 1.17 ± 2.6 mg/L, p-value = 0.001). A significantly positive relationship was found between serum cystatin C levels and creatinine and high-sensitivity C-reactive protein (hs-CRP) levels in both groups (r-value = 0.288, p-value = 0.005, r-value = 0.143, p-value = 0.007, respectively). In multivariate logistic regression analysis, serum cystatin C level found to be a significant predictor for the presence of iCAE (OR: 0.837, CI: 95% (0.341 - 1.637), p-value = 0.013). Receiver operating characteristic (ROC) analysis determined that a cystatin C value < 1.02 mg/L had a sensitivity of 56% and a specificity of 78% for the prediction of ectasia.

Conclusion

We conclude that cystatin C independently can be a useful predictor for the presence of iCAE.

## Introduction

Isolated coronary artery ectasia (iCAE) is characterized as a localized or diffuse, non-occlusive dilatation of epicardial coronary arteries in the diameter of a coronary artery segment of 1.5-fold normal size, considering as such the adjacent non-dilated segments [1-2].

The pathophysiological mechanism of iCAE has become an important research topic because of its similar mortality rate compared to patients with multivessel coronary heart disease [3].

Unfortunately, there is still limited information on the underlying biological process. Previous trials have suggested that several pathways, including those involved in the response to wounding, lipoprotein remodeling, platelet activation, and blood coagulation, may differ between healthy volunteers and patients with iCAE [3-5].

The main underlying pathophysiologic process in iCAE could be associated with enzymatic degradation of the extracellular matrix (ECM) of the media, especially the overexpression of matrix metalloproteinases (MMPs), which could result in excessive expansive arterial remodeling [6].

Cystatin C is an endogenous secretory protein that inactivates the proteolytic enzymes, such as cysteine proteinases. We believe that cysteine proteases, which predominate because of the reduction or inhibition in cystatin C expression, may take a place in the inflammatory and dilating process of iCAE due to its elastolytic and collagenolytic activities [7-8]. There is also growing evidence that the reduction or inhibition in cystatin C expression correlates with dilating vascular disease [9-10].

The present analysis aimed to assess the value of cystatin C as a predictor of ICAE and to further our current understanding of the pathogenesis of iCAE.

## Materials and methods

Study design

The protocol was approved with the registration number of 2011-KAEK-25 2015/20-07 by the local ethics committee in accordance with the ethical principles for human investigations, as outlined in the second Declaration of Helsinki, and conducted at a tertiary referral cardiology hospital. All patients provided written informed consent prior to participating.

Patient selection

In total, 79 individuals were included in this cross-sectional study. The study group included 47 patients with iCAE without any stenotic lesions and the control group consisted of 32 age-and gender-matched subjects who proved to have normal coronary angiograms.

Exclusion criteria

Aortic or cerebral aneurysm;

Patients with high troponin due to any causes (including acute coronary syndrome (ACS), acute and chronic heart failure, pulmonary embolism, obstructive coronary artery disease (CAD) who had coronary stenotic lesions of > 20%, etc.;

History of cardiovascular disease including previous myocardial infarction, coronary artery surgery, arrhythmias, or moderate/severe valvular disease);

Chronic systemic disease (including chronic lung disease, hypertension, diabetes mellitus, obstructive sleep apnea, known malignancy, thyroid dysfunction, active infection or sepsis, chronic kidney disease, stroke, chronic inflammatory diseases, previous stroke, liver disease, rheumatological diseases, etc.)

Coronary angiography assessment

Coronary angiography was performed with a femoral approach (with the Judkins technique) without the use of nitroglycerin, adenosine, or a calcium channel blocker by using Siemens Axiom Artis dFC system (Siemens Medical Solutions, Erlangen, Germany). The results were evaluated by at least two independent interventional cardiologists.

Analysis of cystatin C

All blood samples were collected after a fasting period of eight hours from a large antecubital vein without interruption of venous flow and collected in 13 x 100 mm biochemical tubes with a yellow cap containing gel (BD vacutainer) and 2.0 mL purple top tubes coated with K3EDTA (BD vacutainer). For measurement of cystatin C, serum was separated after centrifugation at 1,500 g for 10 minutes and stored at -80℃ until analysis, and all samples were processed simultaneously. Cystatin C levels were measured with an auto-analyzer (Architect® c16000, Abbott Laboratories, Chicago, IL, USA). The sensitivity of the assay kit was 0.05 ng/mL with a limit of detection of 0.3 ng/mL. The kit also has intra-assay and inter-assay coefficient of variation of < 10% and < 12%, respectively, and used the principle of particle-enhanced turbidimetric immunoassay (PETIA) to assay the concentration of cystatin C. Results were presented as mg/L.

Statistical analysis

The Statistical Package for Social Sciences (SPSS) software package, version 26.0 (IBM SPSS Statistics, Armonk, NY) was used to perform all data analyses. The distribution pattern of the variables was analyzed using the Kolmogorov-Smirnov test. Continuous data are presented as mean ± standard deviation, or median and interquartile range, according to the distribution pattern of the variables. The student’s t-test was used to compare parametric continuous variables, and the Mann-Whitney U test was used to compare nonparametric continuous variables. Categorical variables were compared using the Chi-square test, the results of which are presented as percentages. The correlations were assessed by performing Pearson's correlation test. The effects of different variables on iCAE were determined with multivariate analysis. In all statistical analyses, a p-value of < 0.05 was considered significant.

## Results

Demographic, clinical, and laboratory characteristics of subjects

**Figure 1:**
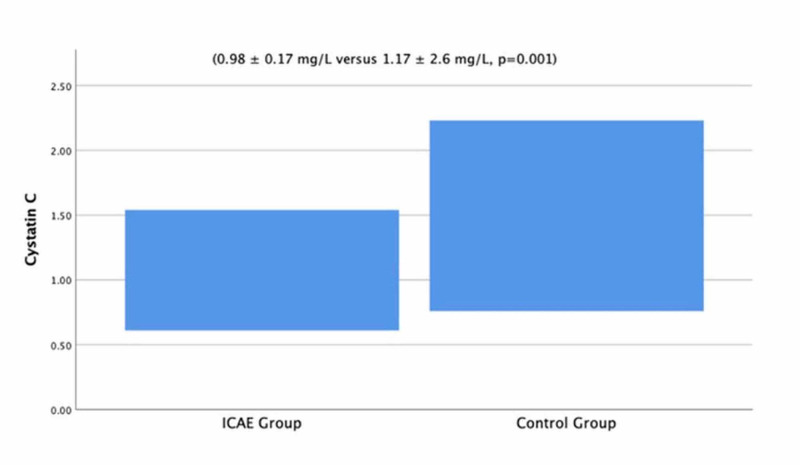
Serum cystatin C concentrations of the patients with isolated coronary artery ectasia and control group iCAE: isolated coronary artery ectasia

The groups did not significantly differ with regard to baseline clinical and laboratory characteristics. The comparison is summarized in Table 1. Serum cystatin C concentrations were significantly lower in patients with iCAE compared with the control group (0.98 ± 0.17 mg/L versus 1.17 ± 2.6 mg/L, p = 0.001) (Figure 1).

**Table 1:** Baseline Characteristics and Laboratory Findings of the Study Patients ALT: alanine aminotransferase; AST: aspartate aminotransferase; CRP: C-reactive protein; TSH: thyroid-stimulating hormone,

Variables	Patients with isolated CAE (n = 47, 65%)	Control group (n = 32, 35%)	p-value
Age (years)	55.9 ± 11.5	57.8.1 ± 9.6	0.29
Sex (n, %) males	24 (51%)	16 (50%)	0.28
Body mass index (kg/m²)	24.7 ± 3.1	25.2 ± 4.2	0.87
Fasting glucose (mg/dL)	99.8 ± 10.7	96.2 ± 9.5	0.89
Creatinine (mg/dL)	0.76 ± 0.5	0.81 ± 0.2	0.69
Triglyceride (mg/dL)	159 ± 29	151 ± 32	0.74
TSH (µIU/mL)	1.37 ± 0.8	1.34 ± 0.6	0.57
Sodium (Na) (mmol/L)	139 ± 10	140 ± 9	0.58
Potassium (K) (mmol/L)	4.7 ± 0.7	4.3 ± 1.1	0.17
AST (U/L)	27 ± 8	26 ± 4	0.61
ALT (U/L)	26 ± 14	25 ± 9	0.32
CRP (nmol/L)	37 ± 18	39 ± 24	0.45
Leukocytes (10^9/L)	8.2 ± 5.5	8.7 ± 6.1	0.47
Hemoglobin (g/dL)	13.9 ± 2.7	13.5 ± 2.2	0.33
Platelets (10^9/L)	285 ± 95	271 ± 76	0.84

Correlation analyses of factors relating to coronary ectasia and cystatin C

**Figure 2:**
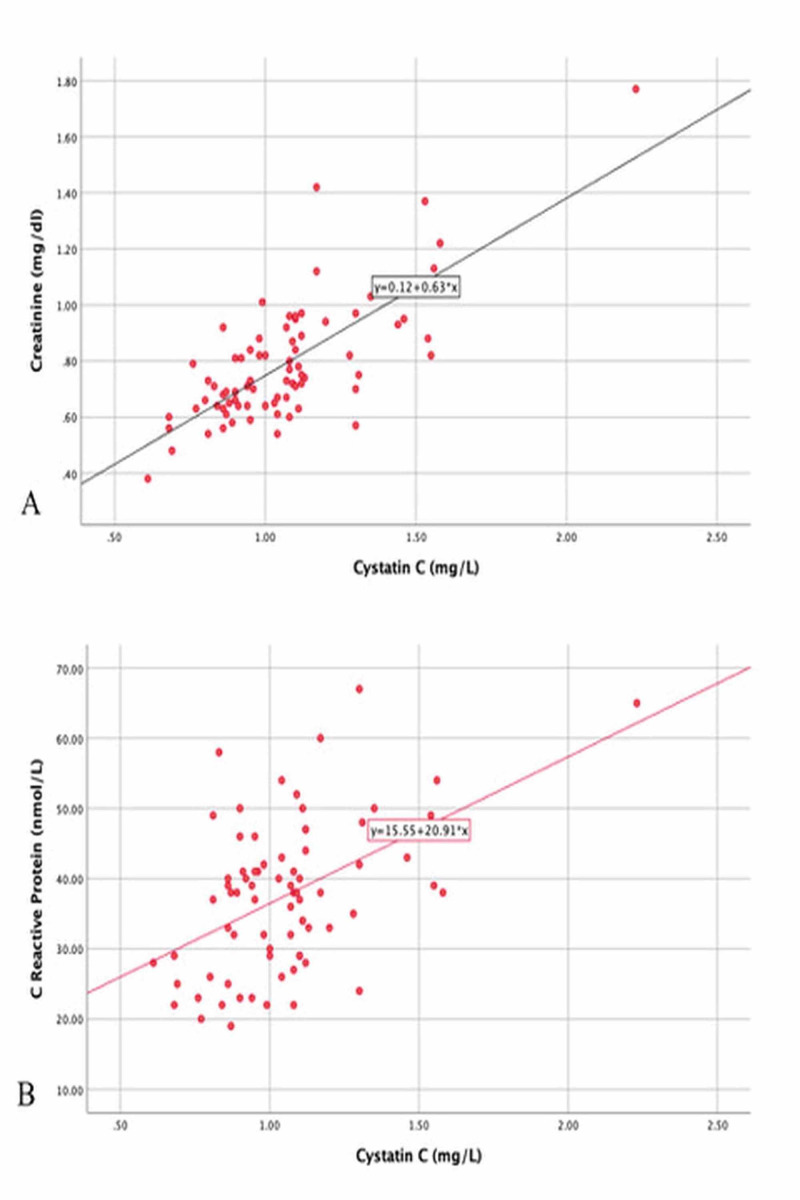
The correlation between cystatin C and creatinine (A) and C-reactive protein (B)

In correlation analysis, a significantly positive relationship was found between serum cystatin C levels and creatinine and hs-CRP levels in both groups (r = 0.738, p = 0.001 and r = 0.499, p = 0.001, respectively) (Figure 2). There was no correlation with the other parameters.

Regression analysis for assessment of the presence of ectasia

Multivariate logistic regression analysis was performed to investigate independent correlates of iCAE and other parameters. In multivariate logistic regression analysis, the serum cystatin C level was found to be a significant predictor for the presence of iCAE (OR: 0.837, 95% CI: 0.341 - 1.637, p = 0.013) (Summarized in Table 2).

**Table 2:** Identification of the Predictors for Isolated Coronary Artery Ectasia with Multiple Regression Analysis CI: confidence interval; CRP: C-reactive protein; OR: odds ratio; TSH: thyroid-stimulating hormone

Variables	OR	Coefficient 95% CI	P-value
Age	0.967	0.859 – 1.067	0.45
Creatinine	0.926	0.786 – 1.226	0.23
CRP	0.948	0.839 – 1.054	0.26
TSH	1.022	0.981 – 1.049	0.64
Cystatin C	0.837	0.541 – 1.537	0.013

ROC analysis to determine the optimal cut-off of cystatin C for prediction of ectasia

**Figure 3:**
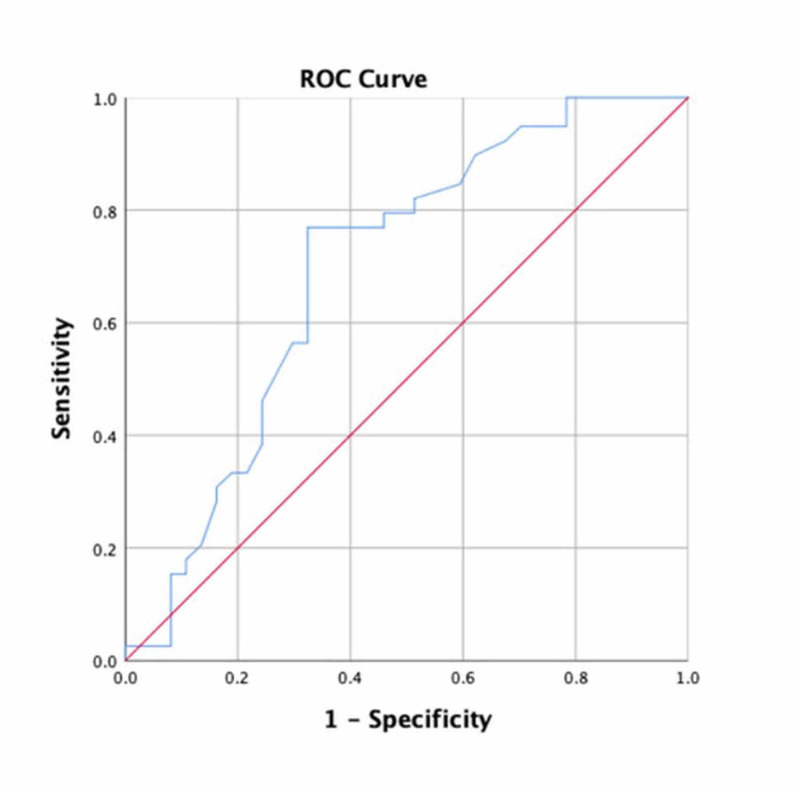
Receiver operating characteristic (ROC) analysis to determine the association between isolated coronary artery ectasia and cystatin C Cut-off value of < 1.02 mg/L demonstrated a sensitivity of 56% and a specificity of 78% (area under the curve: 0.67, p = 0.001, 95% CI: 0.55 – 0.78).

The area under the ROC curve of cystatin C for the prediction of ectasia in all patients was 0.67 (p = 0.001, 95% CI: 0.55 - 0.78). A cystatin C value < 1.02 mg/L had a sensitivity of 56% and a specificity of 78% for the prediction of ectasia (Figure 3).

## Discussion

**Figure 4:**
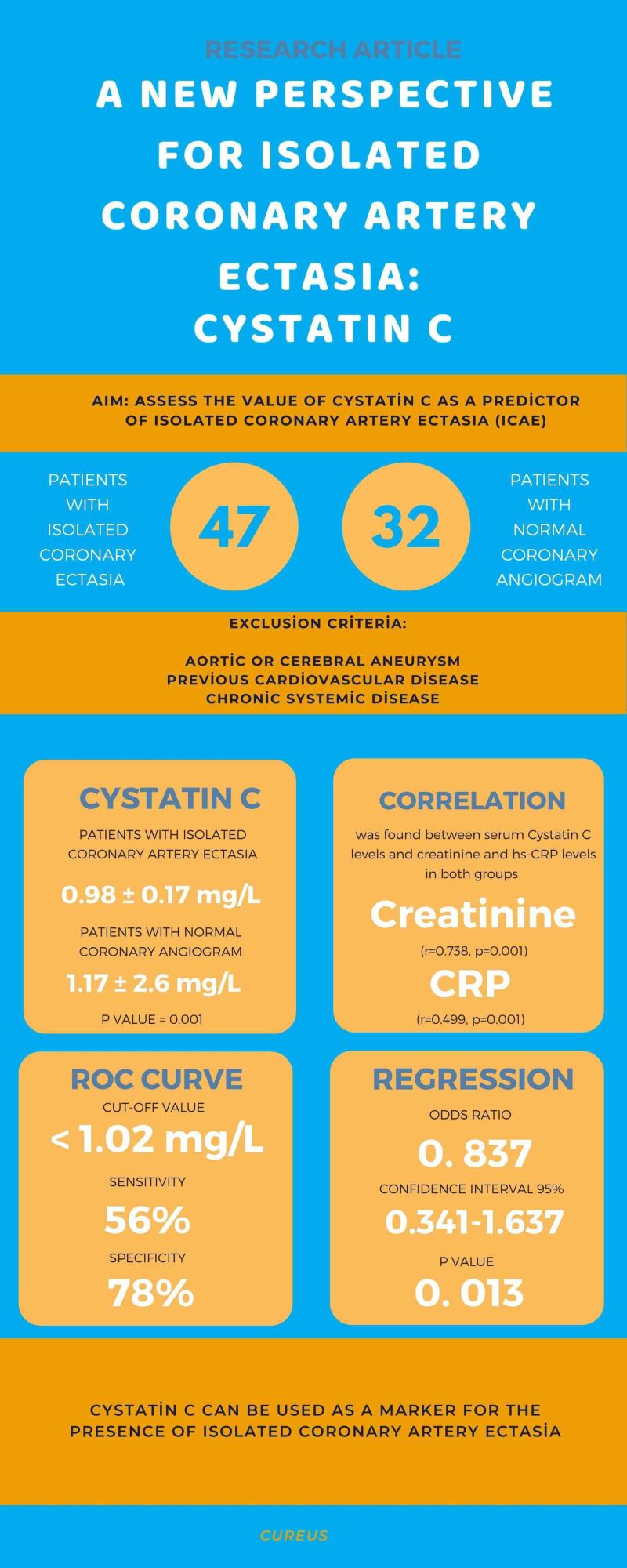
Infographic demonstration of the study

The main finding of this present study is that the patients with iCAE had a lower cystatin C level compared to the individuals with normal coronary artery. Furthermore, cystatin C was associated with the presence of coronary ectasia, and cystatin C levels < 1.02 mg/L had a sensitivity of 56% and a specificity of 78% for the prediction of ICAE. The infographical abstract of the study was seen in Figure 4.

The exact pathogenesis of ectasia has not been clearly identified. The main hypothesis of this study was that cystatin C deficiency may result in vascular enlargement with an imbalance in favor of MMP. The results of this study support the view of increased MMP activity in iCAE pathophysiology. This has been demonstrated in vivo studies, but not enough clinical trials have been performed yet [11-13].

Cystatin C is known as a secretory protein that inactivates the proteolytic enzymes, such as cysteine proteinases (cathepsin) and serine proteinase (neutrophil elastase, plasminogen activator, tryptase kinase, plasmin, etc.). Thus, cystatin C may have a crucial role in protein catabolism during ECM remodeling [14-15]. Overexpression of cysteine proteases may take a place in the inflammatory and dilating process due to its elastolytic and collagenolytic activities.

Showing lower cystatin C levels in aortic aneurysmal tissues in immunohistochemical studies caused plasma cystatin C levels to be investigated in patients with dilating vascular disease.

Firstly, Sukhova et al. examined the expression of the potent elastases cathepsins S and K in human atheroma [16]. They found that normal arteries contained little or no cathepsin K or S and also demonstrated that extracts of atheromatous tissues had approximately two-fold greater elastase-specific activity than extracts of uninvolved arteries, mostly due to cysteine proteases [16].

Similarly, Aoki et al. investigated the role of cysteine proteases in cerebral aneurysms with animal models. A cerebral aneurysm was created surgically in mice, and it was demonstrated by immunohistochemistry methods that after three months of follow-up, the levels of cathepsin B, S, and K in the aneurysmal tissues increased and the cystatin C level decreased. After the use of NT-2300, which is a cysteine protease inhibitor, it was observed that cathepsin activity and collagenase 1 and 4 activity in the aneurysm wall decreased while the level of elastin increased [17].

Dogan et al. found higher plasma levels of MMP-3 and MMP-9 in CAE patients compared to CAD patients and those with normal coronary arteries [18]. Luttun et al. analyzed the role of MMP-9 and MMP-12 in atherosclerosis and media destruction using the apolipoprotein deficient (apoE−/−) mice model [19]. They found that MMP-9 or MMP-12 deficiency protected against atherosclerotic media destruction and ectasia. However, in our view, the role of ECM destruction in iCAE pathogenesis may be the last domino stone.

There are a few studies investigating the cutoff for the cystatin C level as a prognostic indicator in process of various cardiovascular conditions. Budano et al. found that a cystatin C level > 1.4 mg/L as a predictor for long-term adverse events before coronary angiography had a sensitivity of 59%, a specificity of 83%, and a negative predictive value of 98% [20]. Mao et al. also investigated the association of cystatin C with metabolic syndrome (MetS) and cardiovascular outcomes in non-ST-segment elevation ACS with preserved renal function [21]. They found that the predictive values of cystatin C for MetS and major adverse cardiovascular events were 1.01 mg/L and 0.87 mg/L, respectively. From another perspective, Wang et al. found that the level of cystatin C had a linear trend with the risk of ischemic stroke (P for trend = .0049) [22]. We showed that a cystatin C level < 1.02 mg/L had a sensitivity of 56% and a specificity of 78% for the prediction of ectasia.

We encountered a positive relationship between cystatin C levels and hs-CRP, which is a classic indicator of inflammation and creatinine level, which represents a reduced glomerular filtration rate (GFR). Although the correlation between hs-CRP and cystatin C was found to be statistically significant, very little correlation was observed in real terms. This may be because of the patients with high hs-CRP levels who were excluded from the study. Therewith, our results were complementary to previous studies [23-24].

When we compare these results with our own study, we see that our results are compatible with the literature. However, the first question that may come to mind as a criticism at the end of these studies is why the destruction is only in vascular tissues. When we review the literature from this point of view, unfortunately, despite the developments in preclinical and clinical studies, we have not yet found a credible answer to this question. However, another hypothesis is that cystatin C expression in vascular tissues is higher compared to other tissues, and it is likely that there is another protective mechanism against cystatin C-like elastolytic effects in other tissues. In any case, it is very clear that this point will remain the focus of the researchers.

It is unclear whether cystatin C deficiency is acquired congenitally or later. It is also not clear at what age the first coronary enlargement first appeared. Therefore, we recommend performing an age-related subgroup analysis in much larger population studies.

This study has some strengths and limitations that are worth noting. One of the strengths is that, in other models of cystatin C, a deficiency in aortic and cerebral aneurysm has been demonstrated and the patient group mentioned was excluded in our study to obtain reliable results. Another one is that, in recent years, many studies related to iCAE have been conducted and many cytokines that may be associated with iCAE have been identified. The feature that distinguishes this study from them is that the results of this study may shed light on the pathogenesis of iCAE. The first limitation is that coronary artery specimens are optimal for studies; however, they are difficult to collect from patients. Thus, blood samples were used in the present study. Secondly, the patients enrolled in our study were candidates for coronary angiography. Thus, our findings may provide indirect evidence for the general population. Third, we used a single baseline measurement for cystatin C.

## Conclusions

According to the results of our study, we can suggest that serum cystatin C can be used as a marker for the presence of coronary ectasia. ICAE, intracranial aneurysm, and abdominal aortic aneurysm, which are different forms of dilated vascular diseases. All these phenomena can be caused by dysregulated expression of proteases and their endogenous inhibitors. We also recommend that a patient with any of these phenomena should be systematically evaluated for other dilated vascular diseases.
